# Effect of Patient Factors on Portal Vein and Hepatic Contrast Enhancement at Computed Tomography Scan With Protocol Combining Fixed Injection Duration and Patients’ Body Weight Tailored Dose of Contrast Material

**DOI:** 10.7759/cureus.29283

**Published:** 2022-09-18

**Authors:** Hui Ye

**Affiliations:** 1 Department of Medical Imaging, Sun Yat-Sen University Cancer Center, Guangzhou, CHN

**Keywords:** liver neoplasms, fixed duration, linear regression analysis, portal vein, contrast media, ct (computed tomography) imaging

## Abstract

Introduction

Fixed injection duration with patients’ body weight tailored dose of contrast material was recommended as the practical scan protocol in multiphasic contrast-enhanced abdominal computed tomography (CT). This study evaluated the effect of the demographic variables on portal vein and hepatic contrast enhancement in hepatic arterial phase (HAP), aiming to reduce the patient-to-patient variability and optimize the HAP images.

Methods

This retrospective analysis included 87 patients who underwent abdominal enhancement multiphase CT from April to June 2022. All the patients were examined using protocol combining fixed injection duration and patients’ body weight tailored dose of contrast material. Univariate and multivariate linear regression analyses were performed between all patient characteristics and the contrast-enhanced CT number of portal vein and hepatic parenchyma during HAP.

Results

Univariate linear regression analysis demonstrated statistically significant correlations between the CT number of hepatic parenchyma, and the body mass index (BMI), body surface area (BSA), and total body weight (TBW) (all P < 0.001) during HAP. However, multivariate linear regression analysis showed that the BMI or BMI and age were of independent predictive values (P < 0.001). Also, only the age was independently and negatively related to the CT number of portal vein enhancement during HAP (r = 0.240, P < 0.05) according to univariate linear regression analysis.

Conclusions

Univariate linear regression analysis revealed a significant inverse correlation between portal vein CT value and age. By multivariate linear regression analysis, only the BMI and age were significantly correlated with liver parenchymal enhancement, while gender, TBW, BSA, and HT were not.

## Introduction

Triple-phase helical CT of the liver was widely used in enhanced CT scan of the liver. Among the three phases, hepatic arterial phase (HAP) was important in enhanced CT scan by maximizing liver/lesion differences in attenuation [1]. Late HAP images improved the detectability of hepatocellular carcinoma and liver metastases, especially of smaller lesions [2-3].

Bolus tracking and test bolus are commonly used methods to determine the scan timing of CT images. Permitting more efficient use of contrast medium and a shorter imaging time, the bolus tracking application is more widely used to determine the acquisition timing of CT angiographic studies [4-8]. Some studies showed that the timing of bolus triggering was more accurate and that the dose of contrast material required in bolus triggering was less compared with standard administration [7-8]. However, no uniform formula has been applied for the calculation of an imaging delay in bolus tracking method. In addition, some studies showed that the value of automatic bolus tracking in late-arterial and portal-venous phase imaging of the liver with a multislice CT scanner is limited [9-10]. There has been a lot of research trying to improve the scan triggering technique [11-14], but these applications were pilot studies, and their superiority needs to be verified.

Empirically fixed delay from contrast injection was still the main method of triple-phase helical CT in our daily examinations because of its relatively simple workflow and lower radiation dose. The use of fixed injection duration of patients’ body weight tailored dose of contrast material was recommended as ultimately optimal but practical injection/scan protocol by Ichikawa et al. [15]. According to them, the portal venous structures are moderately contrast-enhanced but hepatic veins are not contrast-enhanced in images of optimal scan timing of HAP. However, due to the impact of individual and contrast injection factors, it is difficult to grab these optimal scan timing HAP images, as sometimes the portal venous structures are not contrast-enhanced or sometimes hepatic veins are contrast-enhanced. In addition, the patient-to-patient variability of liver and vascular enhancement was obvious.

This retrospective study would be helpful in exploring a better multiphasic contrast-enhanced CT protocol of the liver. After finding the factors that have a significant impact on portal vein and hepatic parenchyma enhancement during HAP, this injection and scan protocol will probably be improved by adjusting the protocol according to these factors.

## Materials and methods

This retrospective analysis included 87 patients who underwent abdominal enhancement multiphase CT from April to June 2022 at our institute, excluding patients with incomplete records of height (HT) and weight, serious cirrhosis, and serious fatty liver, and patients after partial hepatectomy. Among the 87 patients, there were 45 men and 42 women, with an age range of 26 to 85 years (mean: 56.74 years) (Table 1). In these 87 patients, the clinical diagnoses were lung cancer (n = 49), nasopharyngeal carcinoma (n = 14), breast cancer (n = 3), colorectal cancer (n = 4), cervical cancer (n = 1), liver lesions (n = 2), esophageal cancer (n = 2), thyroid nodule (n = 1), oropharyngeal cancer (n = 1), pancreatic cancer (n = 1), prostate cancer (n = 1), gastric cancer (n = 1), renal carcinoma (n = 1), ovarian cancer (n = 1), malignant peritoneal mesothelioma (n = 1), urachal anomalies (n = 1), neuroendocrine carcinoma (n = 1), and lung neoplasm (n = 2). Approval from the Institutional Review Board of Sun Yat-Sen University Cancer Center was obtained for this retrospective study on August 23, 2022 (approval number: B2022-427-01).

**Table 1:** Patient characteristics. TBW, total body weight; HT, height; BSA, body surface area; BMI, body mass index

	Minimum	Maximum	Mean
Age	26.00	85.00	56.74 ± 11.73
TBW	34.00	96.00	58.42 ± 11.57
HT	140.00	185.00	162.62 ± 8.36
BSA	1.16	2.20	1.62 ± 0.19
BMI	15.11	28.37	21.91 ± 2.95

Using a 128-detector CT scanner (Brilliance iCT; Philips Healthcare, Cleveland, Ohio), contrast-enhanced multidetector CT studies were performed with the patient in the supine position during a single breath-hold. We scanned craniocaudally from the top to the lower end of the liver. The CT scanning parameters were 0.5-second rotation scan, 128×0.625-mm collimation, 0.993 helical pitch, 158.9mm/second table speed, 120-kVp tube voltage, using Z-DOM dose modulation. The scanning time varied from 1.9 to 2.4 seconds.

We inserted a 22-gauge catheter into an antecubital (in most patients) or cubital vein and injected the contrast material with Iopamidol 370 (37g I/100mL) 1.2 mL/kg with a power injector (XD 8006, Ulrich Gmbh & Co.KG, Buchbrunnenweg, Germany) in 29-31 seconds. All the contrast media was warmed with body temperature (about 37 degrees C) before administration, reducing its viscosity and increasing the efficiency of delivering high-viscosity contrast media through small-bore catheters. A total of 30 mL of saline flush was injected at the same rate as the contrast material injection at the end of the duration.

All the patients underwent abdomen plain CT and contrast-enhanced CT including HAP, hepatic parenchymal phase (HPP), and equilibrium phase (EP). Scan start time after the beginning of injection of contrast material for each phase was 35 seconds for HAP, 65 seconds for HPP, and 3 minutes for EP.

In the liver, three small circular regions of interest (ROI) with an area of approximately 50mm^2^ were selected respectively from the left, right anterior, and posterior hepatic lobes, being at a distance of about 1cm from the edge of the liver and avoiding blood vessels, focal hepatic lesions, bile ducts, calcifications, and artifacts. CT mean numbers of the three ROIs were calculated and recorded as liver parenchyma CT values. The CT numbers were also measured by an ROI within the main portal vein.

Patient’s gender, age, total body weight (TBW), HT, body mass index (BMI), and body surface area (BSA) were thought to be the factors of contrast enhancement in CT imaging according to previous articles [16-18]. The gender, age, TBW, and HT of the patients were recorded, and the BMI and BSA were calculated. Estimated BSA (in square meters) was calculated using the following equation: BSA = 0.007184 times (H)^0.725^×(W)^0.425^, where H is HT (in centimeters) and W is TBW (in kilograms) [19]. The BMI was defined as TBW (in kilograms) divided by the square of the HT (in meters).

Univariate and multivariate linear regression analyses were performed between all patient characteristics and the contrast-enhanced CT number of portal vein or hepatic parenchyma during HAP. Variables with p-values less than 0.05 were considered statistically significant. The Statistical Package for Social Sciences software (SPSS for Windows, Version 26.0, IBM Corp., Armonk, NY) was used for statistical computing.

## Results

As presented in Table 1, of the 87 patients, 45 were men and 42 were women. their age ranged from 26 to 85 years (mean: 56.74 ± 11.73 years), the TBW from 34.0 to 96.0 kg (mean: 58.4 ± 11.6 kg), HT from 140.0 to 185.0 cm (mean: 162.6 ± 8.4 cm), BSA from 1.2 to 2.2 m^2^ (mean: 1.6 ± 0.2 m^2^), and BMI from 15.1 to 28.4 kg/m^2^ (mean: 21.9 ± 3.0 kg/m^2^).

Among the 87 patients, there were no patients’ HAP images with contrast-enhanced hepatic veins, indicating the HAP scan timing, which was 35 seconds after the beginning of injection of contrast material, which cannot be considered as too late in daily clinical practice according to the visual confirmation of HAP scan timing suggested by Ichikawa et al. [15].

**Figure 1:**
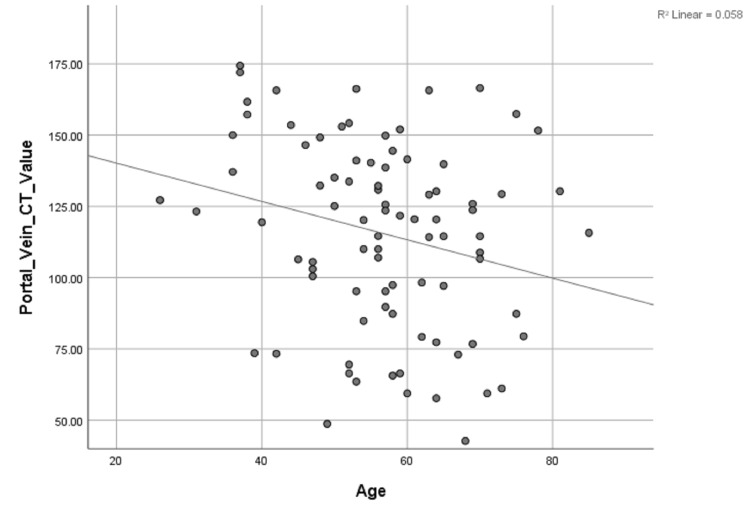
Scattergrams of the relationship between portal vein CT value and the patient age. Unitary linear regression model: y=108.411-1.415x. y, portal vein CT value; x, age; R, related coefficient

Univariate linear regression analysis only revealed a significant inverse correlation between portal vein CT value and age (r = 0.240, P < 0.05) (Table 2, Figure 1).

**Table 2:** Effect of age, TBW, HT, BMI, and BSA on portal vein CT value during HAP. R, related coefficient; TBW, total body weight; HT, height; BMI, body mass index; BSA, body surface area; HAP, hepatic arterial phase; Sig., significant

Model	R	R square	Durbin-Watson	F	Sig.
Age	0.240	0.058	1.927	5.216	0.025
TBW	0.004	0.000	1.967	0.001	0.974
HT	0.004	0.000	1.965	0.001	0.970
BMI	0.014	0.000	1.972	0.016	0.898
BSA	0.003	0.000	1.966	0.001	0.981

**Figure 2:**
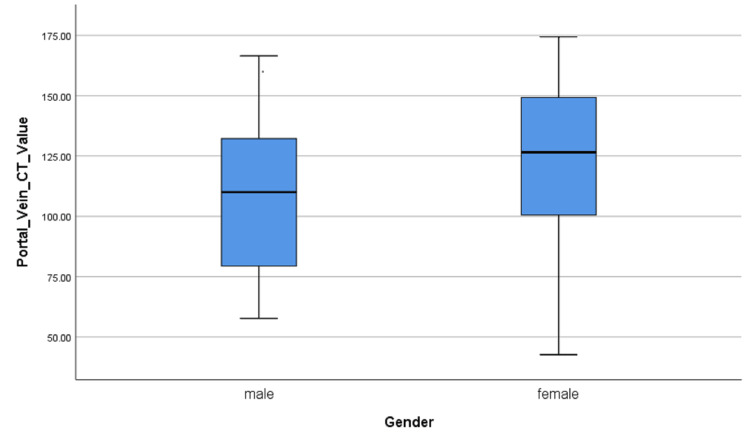
The box-and-whisker plot showing portal vein CT value for men and women.

According to Levene’s test for equality of variances and t-test for equality of means, the variance between the male and female groups of data is uniform and there was no significant difference in the portal vein CT value between men and women (109.59 ± 31.895 vs 121.76 ± 33.006 HU, P = 0.084) (Table 3, Figure 2).

**Table 3:** Levene’s test for equality of variances and t-test for equality of means. df, degree of freedom; Sig., significance

	Levene’s test for equality of variances			t	df	Sig. (two-tailed)	Mean difference
		F	Sig.				
Portal vein CT value	Equal variances assumed	0.053	0.818	-1.748	85.000	0.084	-12.166
	Equal variances not assumed			-1.746	84.091	0.084	-12.166

**Figure 3:**
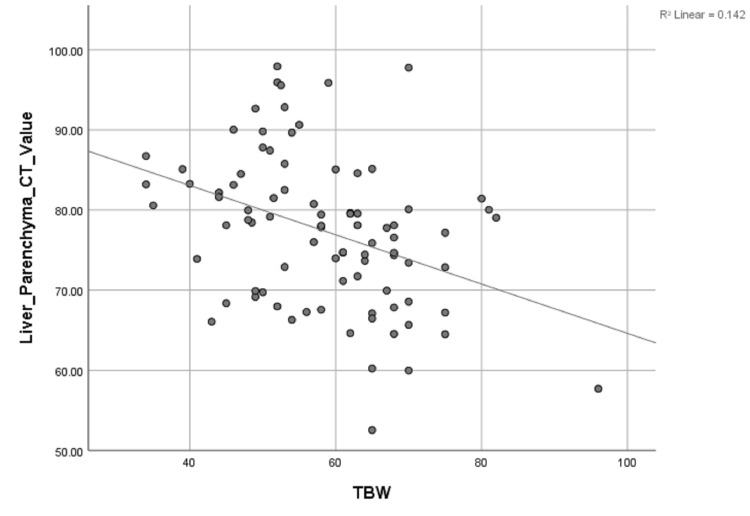
Scattergrams of the relationship between liver parenchyma CT value and TBW. Unitary linear regression model: y=108.411-1.415x. y, liver parenchyma CT value; x, total body weight; TBW, total body weight; R, related coefficient

**Figure 4:**
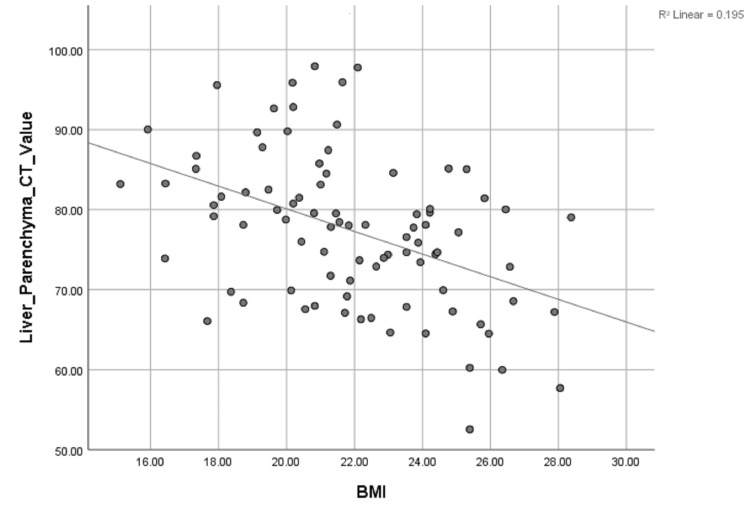
Scattergrams of the relationship between liver parenchyma CT value and BMI. Unitary linear regression model: y=108.411-1.415x. y, liver parenchyma CT value; x, body mass index; BMI, body mass index; R, related coefficient

**Figure 5:**
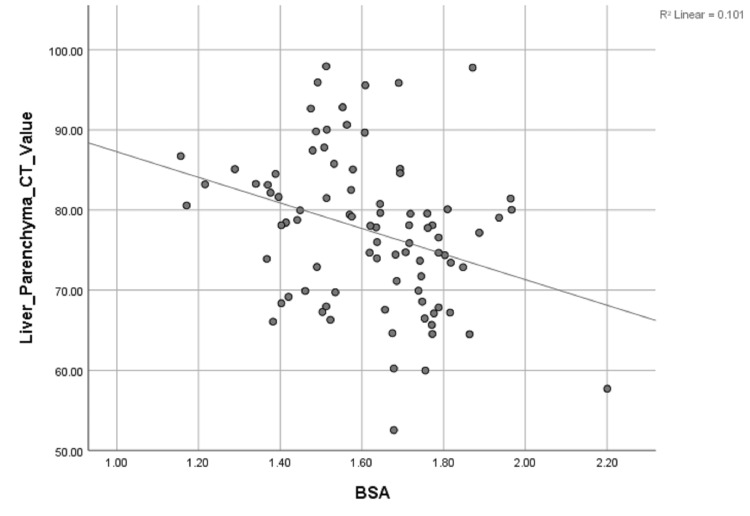
Scattergrams of the relationship between liver parenchyma CT value and BSA. Unitary linear regression model: y=108.411-1.415x. y, liver parenchyma CT value; x, body surface area; BSA, body surface area; R, related coefficient

Univariate linear regression analysis revealed a significant inverse correlation between liver parenchyma CT value, and the TBW (r = 0.376), BSA (r = 0.317), and BMI (r = 0.442), (P < 0.01 for all) (Table 4, Figures 3-5).

**Table 4:** Effect of age, TBW, HT, BMI, and BSA on liver parenchyma CT value during HAP. R, related coefficient; Sig., significant; TBW, total body weight; BMI, body mass index; BSA, body surface area; HT, height; HAP, hepatic arterial phase

Model	R	R square	Durbin-Watson	F	Sig.
Age	0.181	0.033	1.361	2.882	0.093
TBW	0.376	0.142	1.568	14.038	0.000
BMI	0.442	0.195	1.612	20.635	0.000
BSA	0.317	0.101	1.512	9.506	0.003
HT	0.141	0.020	1.398	0.722	0.193

**Figure 6:**
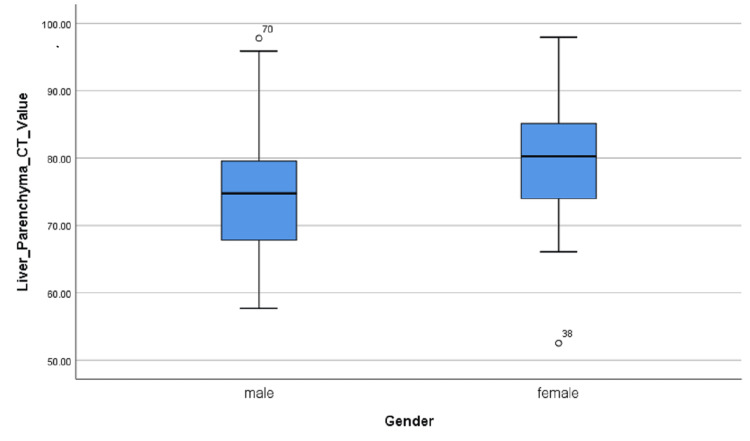
The box-and-whisker plot showing liver parenchyma CT value for men and women. Sig., significance

According to Levene’s test for equality of variances and t-test for equality of means, the variance between the male and female groups of data is uniform and there was a significant difference in the liver parenchyma CT value between men and women (75.21 ± 9.194 vs 79.75 ± 9.268 HU, P = 0.024) (Table 5, Figure 6).

**Table 5:** Levene’s test for equality of variances and t-test for equality of means. df, degree of freedom; Sig., significance

	Levene’s test for equality of variances			t-test for equality of means	df	Sig. (two-tailed)	Mean difference
		F	Sig.	t			
Liver parenchyma CT value	Equal variances assumed	0.025	0.874	-2.291	85.000	0.024	-4.536
	Equal variances not assumed			-2.290	84.488	0.025	-4.536

However, multivariate linear regression analysis showed that only age and BMI were of independent predictive values for liver parenchyma CT value (P < 0.01) (Tables 6, 7).

**Table 6:** Results of multivariate linear regression analysis of the enhancement of the liver parenchyma during HAP. HAP, hepatic arterial phase; Sig., significant; VIF, variance inflation factor; BMI, body mass index; Std. error, standard error

Model		Unstandardized coefficients		Standardized coefficients	t	Sig.	Collinearity statistics	
		B	Std. error	Beta			Tolerance	VIF
1	(Constant)	108.411	6.888		15.740	0.000		
	BMI	-1.415	0.312	-0.442	-4.543	0.000	1.000	1.000
2	(Constant)	119.452	8.302		14.388	0.000		
	BMI	-1.468	0.305	-0.458	-4.812	0.000	0.994	1.006
	Age	-0.174	0.077	-0.216	-2.268	0.026	0.994	1.006

**Table 7:** Excluded variables of multivariate linear regression analysis of the liver parenchyma CT value during HAP. ^a^Dependent variable: liver parenchyma CT value. ^b^Predictors in the model: constant, BMI. ^c^Predictors in the model:  constant, BMI, and age. "Beta In" values can be treated as possible "Beta" values but not correlation coefficient Sig., significant; TBW, total body weight; HT, height; BMI, body mass index; BSA, body surface area; HAP, hepatic arterial phase

Excluded variables^a^						
Model		Beta In	t	Sig.	Partial correlation	Collinearity statistics
						Tolerance
1	Gender	0.138^b^	1.383	0.170	0.149	0.936
	Age	-0.216^b^	-2.268	0.026	-0.240	0.994
	TBW	0.064^b^	0.306	0.760	0.033	0.219
	BSA	0.066^b^	0.424	0.673	0.046	0.397
	HT	0.046^b^	0.431	0.668	0.047	0.835
2	Gender	0.139^c^	1.428	0.157	0.155	0.936
	TBW	-0.025^c^	-0.121	0.904	-0.013	0.211
	BSA	-0.005^c^	-0.034	0.973	-0.004	0.380
	HT	-0.005^c^	-0.045	0.964	-0.005	0.797

## Discussion

According to previous studies, after using the protocol with contrast material dose determined according to patient weight, aortic peak time, aortic peak enhancement, and period when aortic enhancement is 200 HU were not related to the patients’ TBW [20], maximum aortic and hepatic parenchyma enhancement, time to maximum hepatic enhancement, and the end of HAP were not related to the injection rates [21]. Further research has shown that the peak enhancement times of aorta, main portal vein, and liver parenchyma were approximately 10, 20, and 30 seconds, respectively, after the completion of the injection respectively, regardless of patient weight and injection duration while the dose of contrast material is adjusted to patient weight [22]. However, HAP scanning may be sometimes performed too early or too late with this optimal injection protocol [15]. Since the impact of individual factors was not considered in these studies, analyzing individual factors may be one way to explore a duration providing more uniform scan timing and enhancement. The present research was a retrospective study aiming to evaluate the effect of age, gender, TBW, HT, BSA, and BMI on liver contrast enhancement with scan protocol combining fixed injection duration and patients’ body weight tailored dose of contrast material.

The present study suggests that by univariate linear regression analysis, HT, TBW, BSA, BMI, and gender did not exert a statistically significant effect on portal vein enhancement during the HAP, suggesting that the scan protocol can resolve the interindividual varying transit times of portal vein due to body size, which seemed in agreement with the findings of previous studies [15,20-22].

However, TBW (r = 0.376, P < 0.01), BSA (r = 0.317, P < 0.01), BMI (r = 0.442, P < 0.01) were all significantly related to contrast enhancement of hepatic parenchyma by univariate linear regression analysis, and there was significant difference in the liver parenchyma CT value between men and women. BMI and age have independent predictive values of hepatic parenchyma during the HAP by multivariate linear regression analysis. These results seem to stand in contrast to the results of Awai and Hori [21], who found that maximum aortic and hepatic enhancement was not related to the injection rates (which was perfectly linear correlated to TBW with fixed injection duration with patients’ body weight tailored dose of contrast material). However, in Awai et al.’s study, test injection of contrast material was used to determine start time of the scan, and the start time of the HAP was not recorded and analyzed. In the present study, the scan time started after 35 seconds from the start of the fixed duration for all patients. Therefore, the CT value of hepatic parenchyma was different because of the different rising rate of the CT value caused by the individual factors including TBW, BSA, BMI, gender, and age. It can be inferred from the result above that the start time of the HAP and peak enhancement time of hepatic parenchyma should be delayed in patients with higher TBW, BSA, or BMI, or with older age or being male to reduce the patient-to-patient variability of liver parenchyma.

Studies [23-24] suggest that the enhancement of vessels and parenchymal organs tended to be consistent and adequate regardless of patients’ body weight with BSA-tailored dose of contrast material. Fixed duration injection protocol was also used in these studies, but contrary to the current study, bolus tracking was used for the scanning delay. The object of Yanaga et al.’s study [23] was aortic attenuation and objects of Kondo et al.’s study [24] were aorta and liver attenuation, whereas the current study focuses on portal vein and liver attenuation. In the study by Kidoh et al. [25], fixed duration tailed by TBW was used as well, and the correlation between the CT number per gram of iodine (Δ Hounsfield units/g) and TBW, BMI, BSA, and HT, during HAP were described as strong inverse correlation (r = 0.53, P < 0.001), moderate inverse correlation (r = 0.73, P < 0.001), strongest inverse correlation (r = 0.76, P < 0.001), moderate inverse correlation (r = 0.52, P < 0.001), respectively. Bolus tracking was used for the scanning delay as well, which maybe the reason for the difference with the present study. Since the BMI had the highest Pearson correlation R value with hepatic parenchyma CT value, the present study indicates that the contrast material adjusted by BMI may be a better way to reduce the patient-to-patient variability of parenchyma enhancement during HAP.

 According to Bae’s report, patient body size (weight and HT) and cardiac output (CO) are the key patient-related factors affecting contrast enhancement. The relationship between portal vein or hepatic contrast enhancement and CO was not investigated in the present study. But since CO is perfectly linear correlated to BSA in this study according to the equations in Bae’s study: CO (mL/min) = 25.3 times (H)^0.725^×(W)^0.425^ [16], where H is HT in centimeters and W is TBW in kilograms, the correlation inference can be drawn that there was no significant correlation between portal vein CT value and CO, and that there was a significant inverse correlation between liver parenchyma CT value and CO. This was in accord with the result of Masuda et al.’s study [26], which demonstrated a statistically significant inverse correlation between CO and aortic enhancement per gram of iodine by multivariate linear regression analysis.

In the present study, only the age (r = 0.240, P < 0.05) of the patient was significantly related to contrast enhancement of the portal vein during the HAP by univariate linear regression analysis. The result was in agreement with an earlier study by Sandstede et al. [9], who considered age as a positive factor of delayed contrast material arrival, which might explain the significant decrease in portal vein enhancement in the elderly.

In the present study, there was a significant difference in the liver parenchyma CT value between men and women. This may be caused by the less blood volume in female patients according to Bae’s study [16]. However, there was no significant difference in the portal vein CT value between men and women. This result indicated that the mesenteric blood volumes may be similar between males and females with the same TBW.

Apart from the visual confirmation of HAP scan timing suggested by Ichikawa et al. [15], there were different definitions of an optimal arterial phase. In the study of Sandstede et al., optimal arterial enhancement was defined as 20 ± 30% of hepatic enhancement in the portal venous phase [9]. An earlier study [27] defined optimal arterial phase start when a splenic parenchymal enhancement reach 10 HU, and the end was marked by any decrease of enhancement in the aorta or enhancement in the liver parenchyma of more than 20 HU. Therefore, the factors should be considered differently by different definitions of an optimal arterial phase according to the present study.

Some limitations of our study should be mentioned. First, the study was a retrospective single-center study, and thus more prospective studies are needed to confirm these results. Second, the BSA was calculated according to the DuBois formula. However, there are other formulae such as Mosteller, Gehan, and Haycock formulae, and the study did not compare the effect of BSA calculated by these formulae on the CT numbers in portal vein and hepatic parenchyma. Third, the change in CT number in the portal vein and the hepatic parenchyma from unenhanced images to the HAP images was not calculated. The relationship between patient characteristics and the change in the CT numbers in portal vein and hepatic parenchyma is worth pursuing in further study.

## Conclusions

In conclusion, when hepatic CT was performed under the fixed injection duration with patients’ TBW-adjusted dose of contrast material, the age was the only independent factor with influence on portal vein enhancement during HAP by univariate linear regression logistic analysis. On multivariate linear regression logistic analysis, only BMI and age exhibited a significant correlation with liver parenchymal enhancement during HAP.
